# Airborne Fungal Spore Diversity Assessment Using Culture-Dependent and Metabarcoding Approaches in Bat-Inhabited Natural and Anthropogenic Roosts in Portugal

**DOI:** 10.3390/jof11050360

**Published:** 2025-05-06

**Authors:** Jaqueline T. Bento, Guilherme Moreira, Eugénia Pinto, Priscilla Gomes da Silva, Hugo Rebelo, Joana Mourão, Sofia I. V. Sousa, João R. Mesquita

**Affiliations:** 1 School of Medicine and Biomedical Sciences (ICBAS), University of Porto, 4050-313 Porto, Portugal; jtbento@icbas.up.pt (J.T.B.); gmoreiravet@gmail.com (G.M.); priscidja@caesar.elte.hu (P.G.d.S.); 2 Laboratory of Microbiology, Biological Sciences Department, Faculty of Pharmacy, University of Porto (FFUP), 4050-313 Porto, Portugal; epinto@ff.up.pt; 3 CIIMAR—Interdisciplinary Centre of Marine and Environmental Research, University of Porto, 4450-208 Porto, Portugal; 4 LEPABE—Laboratory for Process Engineering, Environment, Biotechnology and Energy, Faculty of Engineering, University of Porto, 4200-465 Porto, Portugal; sisousa@fe.up.pt; 5 ALiCE—Associate Laboratory in Chemical Engineering, Faculty of Engineering, University of Porto, 4200-465 Porto, Portugal; 6 BIOPOLIS Program in Genomics, Biodiversity and Land Planning, CIBIO, Campus de Vairão, 4485-661 Vairão, Portugal; herebelo@ciencias.ulisboa.pt; 7 CE3C—Centre for Ecology, Evolution and Environmental Changes & CHANGE—Global Change and Sustainability Institute, Departamento de Biologia Animal, Faculdade de Ciências, Universidade de Lisboa, 1749-016 Lisboa, Portugal; 8 National Food Institute, Technical University of Denmark, Kongens Lyngby, 2800 Copenhagen, Denmark; joam@food.dtu.dk; 9 Centro de Estudos de Ciência Animal (CECA), Instituto de Ciências, Tecnologias e Agroambiente (ICETA), Universidade do Porto (UP), 4051-401 Porto, Portugal; 10 Associate Laboratory for Animal and Veterinary Science (AL4AnimalS), 1300-477 Lisboa, Portugal

**Keywords:** fungi, caves, metabarcoding, biodiversity, ecology

## Abstract

Cave environments represent extreme and underexplored ecosystems wherein fungi play a crucial role in nutrient cycling and ecological dynamics. This study provides the first comprehensive assessment of fungal diversity in air samples from caves across Portugal, with six samples from five locations being assessed through culture-dependent and metabarcoding approaches. From the five bat roosts studied, eleven morphologically distinct fungal colonies were isolated, with genera such as *Aspergillus*, *Penicillium*, and *Chaetomium* identified. Concurrently, Oxford Nanopore sequencing of the internal transcribed spacer (ITS) region of fungal rDNA revealed 286 genera, with *Aspergillus*, *Candida*, and *Calyptella* dominating across the sites. Diversity indices and community composition analyses, including Principal Coordinate Analysis (PCoA) and hierarchical clustering, highlighted distinct fungal profiles influenced by site-specific environmental factors and human activity. The data underscores the dual role of fungi in bat roosts as essential decomposers, emphasizing their adaptability to oligotrophic conditions. These findings advance our understanding of subterranean fungal ecology and emphasize the need for targeted conservation efforts to protect cave ecosystems from anthropogenic impacts.

## 1. Introduction

Bat-inhabited natural and anthropogenic habitats, such as caves, mines, and tunnels, are unique microbiological ecosystems shaped by interactions between the environment, bats, and microbes [1]. These environments, with stable microclimates and limited nutrients, support specialized microorganisms. Bats contribute organic matter in the form of guano, corpses, skin, and fur shedding, which serve as nutrients for microbial communities [2]. They also act as vectors and reservoirs, dispersing microorganisms within and between habitats. This dynamic fosters oligotrophic, extremophilic communities that are unique to subterranean environments [3,4].

Bat activity significantly influences fungal diversity, with studies showing a positive correlation between bat abundance and airborne fungi concentration [5]. Bats elevate fungal concentrations in underground environments, particularly during hibernation [6]. Their guano creates conditions that support fungal dispersal and growth, including opportunistic pathogens, which pose risks to both wildlife and human health [4]. Understanding the relationship between bats and fungi is essential for gaining insights into fungal ecology and pathogen transmission.

Bat habitats of anthropogenic origin, such as attics, chimneys, and abandoned buildings, serve as alternative roosting sites for bat populations [7]. These structures often provide stable temperatures, protection from predators, and the seclusion necessary for hibernation or reproduction. The presence of bats in anthropogenic bat roosts creates a unique microbiological environment that is influenced by bat activity [7]. Organic matter, such as guano, urine, and shed fur or skin, accumulates in these spaces, fostering the growth of diverse microbial communities, including fungi, bacteria, and archaea [4].

On the other hand, natural caves are unique and structured ecosystems, with distinct zones influenced by surrounding rock formations, subterranean water, and karst landscapes [8,9]. These subterranean habitats differ markedly from surface environments due to their constant darkness, seasonally stable temperatures, high humidity, and minimal organic matter [10]. The nutrient-poor conditions in bat roosts create challenging living environments, but these spaces also provide a stable climate, which makes them uniquely suited for specialized life forms [11]. Although relatively isolated, bat roosts are influenced by various external factors, including air currents, water flow, and human visitors [12,13].

Together, bat roosts and other subterranean environments represent some of Earth’s most challenging and underexplored ecosystems [11]. Despite the difficulties posed by these extreme conditions, they offer a unique habitat for studying microbial survival, adaptation, and ecology in nutrient-limited environments [8,14].

However, the increasing transformation of natural bat roosts into tourist destinations, often referred to as “show bat roosts”, presents a significant anthropogenic threat to these unique subterranean ecosystems [15,16,17]. Tourism introduces substantial biological disturbances, as microbial propagules are transported by visitors on their clothing, shoes, and skin. This influx leads to biological contamination across multiple cave surfaces and environments, including cave air [17,18], water [17,19], soil [17,20], and even speleothems [17,21]. Additionally, human activity modifies cave substrates [20], enabling the establishment of non-native microbial communities. Opening bat roosts to visitors can also alter their microclimatic conditions, with tourists contributing to increased air temperatures, higher CO_2_ levels [14,22], and introducing organic matter and new microorganisms. These factors collectively support the change of microbial communities within bat roosts [14,23]. Moreover, elevated fungal concentrations, particularly in visitor areas, such as lunchrooms, suggest that human food remnants, like crumbs, may provide a nutrient source that supports foreign microbial populations [24].

Microorganisms, especially fungi, play a crucial role in cave ecosystems, providing energy to cave fauna through decomposition and nutrient cycling within these nutrient-limited environments [13,25]. Fungi are among the dominant organisms in bat roosts, owing to their high spore dissemination rates, ability to colonize diverse substrates, and adaptability to a wide range of pH levels [8,14]. The fungal composition within bat roosts varies depending on the degree of isolation and specific environmental conditions [14]. Some fungal species are essential to cave mycobiota, serving as decomposers and participating in feeding strategies for cave fauna, while others can pose health risks to mammals. For instance, *Pseudogymnoascus destructans* causes white-nose syndrome in bats, and *Histoplasma capsulatum* can lead to systemic histoplasmosis in humans [25,26]. Additionally, certain species of genera, such as *Aspergillus*, *Rhizopus*, and *Fusarium*, are known pathogens in humans, while others like *Alternaria*, *Acremonium*, *Cladosporium*, and *Penicillium* act deleteriously as allergens [13,14].

Fungal spores, frequently found suspended as airborne particles, are prevalent in bat roosts and represent a major component of the microbial diversity within these environments [14,22,25,27]. These spores can also be carried by water, bats, arthropods, and human visitors (e.g., speleologists and researchers). Human visits contribute organic material that supports certain fungi, allowing them to survive and spread, even in previously uncontaminated areas [14,24]. This phenomenon highlights the complex and often delicate balance of microbial communities in subterranean ecosystems, wherein fungi play a vital role as both beneficial and potentially harmful inhabitants.

In this context, the aim of this study was to provide baseline data on fungal diversity across various bat underground roosts in Portugal and to explore their ecological relationships. This research represents the first comprehensive assessment of cave fungi in Portugal. Gaining insight into the ecological roles and interactions of these fungi can help cave managers implement better management practices.

## 2. Material and Methods

### 2.1. Sampling Location

**Figure 1:**
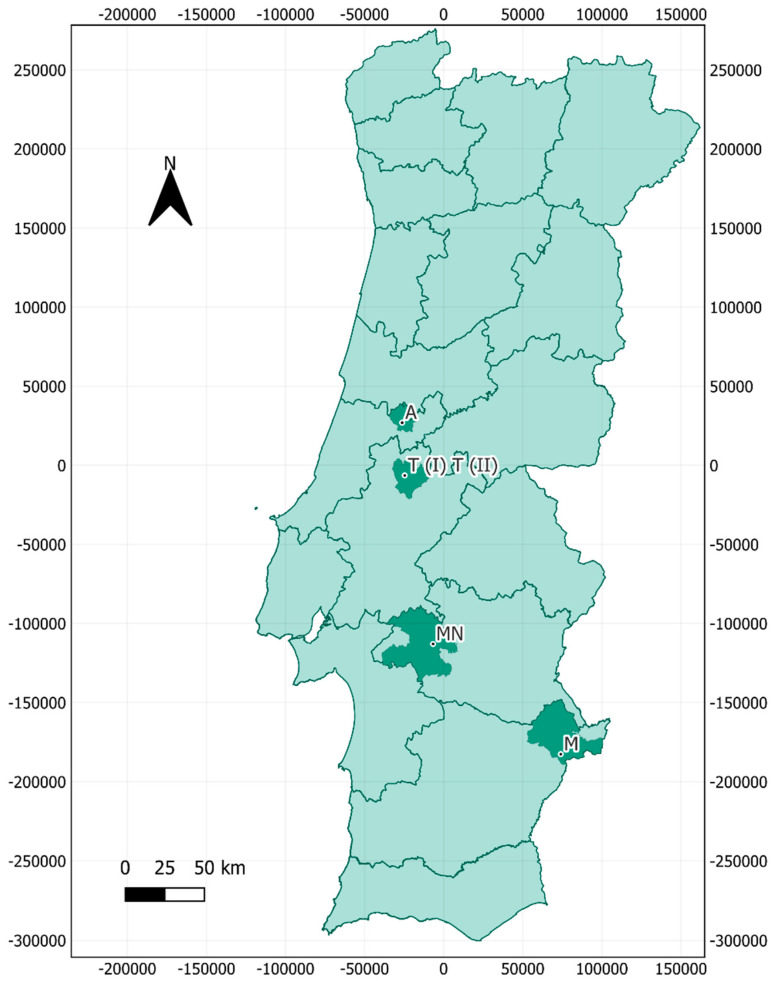
Geographical distribution of air samples collected in bat-inhabited natural and anthropogenic bat roosts in Portugal. Coordinate System: WGS 84/UTM Zone 29N (EPSG: 32629). A: Ansião I; T I: Tomar I; T II: Tomar II; MN: Montemor-o-Novo I; M: Moura I (1)/Moura I (2).

Air sampling took place in July 2022 at five locations across central and southern Portugal, with a total of six samples collected. These locations were one large historical building, two mines, and two caves, located in the municipalities of Montemor-o-Novo (one mine), Ansião (one cave), Tomar (one large historical building (Tomar I) and one cave (Tomar II)), and Moura (two samples taken at different sites within the same mine) (Figure 1). The bat species inhabiting each cavity are presented in Table 1.

For collecting air samples, a Coriolis Compact^®^ air sampler was used in each location, positioned in the center of each cave/building at a height of approximately 1.3 m, except in Moura, in which the samples were collected in the first room (Moura I (2)) and the central room of the mine (Moura I (1)). Sampling was conducted for 60 min, with an airflow rate of 50 L/min. The air samples were collected in a dry sterile Coriolis cone, with 4 mL of sterile Phosphate-Buffered Saline (PBS) added to the collection cones after each sampling. The samples were immediately stored at 4 °C during transportation to the laboratory for further analysis [28]. After collecting each sample, the sampler was cleaned and decontaminated, according to the manufacturer’s instructions. Briefly, a wipe dampened with a surfactant–water solution was used to clean the external parts of the sampler. After that, the sampler was wiped down with a soft cloth to remove any excess solution.

### 2.2. Fungal Culture-Dependent Characterization

The samples were plated onto Sabouraud Dextrose Agar (SDA), supplemented with 100 µg/mL of Penicillin–Streptomycin solution, and incubated at 25 ± 1 °C for 7 to 28 days. The plating process was carried out in triplicate. Colonies that developed were classified into Morphological Taxonomic Units (MTUs) and counted. Pure isolates were obtained on SDA, followed by morphological and genetic analysis. Isolates were examined under a microscope and morphological characterization was carried out using morphological identification keys [29,30,31].

### 2.3. Sequencing of Fungal Isolates

DNA extraction from the fungal samples began by scraping off the mycelium from the cultures and diluting it (10%) in Tissue Lysis (ATL) buffer. The mixtures were incubated at 100 °C for 10 min, followed by centrifugation at 8000× *g* for 5 min. After centrifugation, 140 μL of the supernatant was used for DNA extraction and purification using the QIAamp DNA Mini Kit (Qiagen, Hilden, Germany), according to the manufacturer’s instructions. Automated extraction was carried out using the QIAcube^®^ platform (Qiagen).

The internal transcribed spacer (ITS) region of fungal rDNA was amplified using the primers ITS1 (5′-TCCGTAGGTGAACCTGCGG-3′) and ITS4 (5′-TCCTCCGCTTATTGATATGC-3′) [32]. PCR reactions were performed using a thermocycler (Bio-Rad, Hercules, CA, USA). Reaction mixtures were prepared using the SpeedySupreme NZYTaq 2x Green Master Mix (NZYTech, Lisbon, Portugal), according to the manufacturer’s instructions. The PCR conditions included initial denaturation at 95 °C for 5 min, followed by 35 cycles at 94 °C for 30 s, 55 °C for 30 s, and 72 °C for 1 min, ending with a final extension at 72 °C for 10 min.

Following PCR amplification, the DNA fragments were separated via electrophoresis on 1.5% agarose gels, stained with Xpert Green Safe DNA gel dye (GRiSP^®^, Porto, Portugal). The electrophoresis was run at a constant voltage of 120 V for 25 min. The PCR products were purified using the GRS PCR and Gel Band Purification Kit (GRiSP^®^).

After purification, bidirectional sequencing was performed according to the Sanger method, using the appropriate internal primers for the target gene. The sequences were then aligned with the help of the BioEdit Sequence Alignment Editor v7.1.9 software package, version 2.1v, and compared with the sequences in the NCBI (GenBank) nucleotide database (https://blast.ncbi.nlm.nih.gov/Blast.cgi, accessed on 10 February 2025).

### 2.4. DNA Extraction and Molecular Identification of the Air Samples

Simultaneously, DNA extraction from the air samples was performed using 140 μL of the air samples, according to the instructions on the use of the QIAamp DNA Mini Kit (Qiagen, Hilden, Germany). Automated extraction was carried out using the QIAcube^®^ platform (Qiagen).

PCR, based on the ITS region, followed by gel electrophoresis, were performed as described above. The final DNA concentration was determined using a Qubit 4.0 fluorometer (Thermo Fisher Scientific, Waltham, MA, USA), with the Quant-iT™ 1X dsDNA high sensitivity (HS) Assay Kit (Thermo Fisher Scientific, Waltham, MA, USA), utilizing 2 μL of the DNA elution from each sample as the input.

### 2.5. Oxford Nanopore Sequencing of the ITS

Premium PCR sequencing was conducted using Oxford Nanopore Technology via a PromethION 24 instrument, equipped with an R10.4.1 flow cell. The library preparation utilized the Native Barcoding Kit 96 V14 (SQK-NBD114.96), followed by custom analysis and annotation. The raw FASTQ reads were basecalled in super-accurate mode, using ont-doradod-for-promethion v7.4.12, applying a minimum Q-score of 10, with adapters and barcodes trimmed via MinKNOW.

### 2.6. Taxonomic Classification and Refinement

Initial quality control of the sequencing reads was performed using NanoPlot 1.43.0 [33]. Sequencing adapters and barcodes were removed using Porechop 0.2.4 [34]. To filter the reads, NanoFilt v.2.8.0 [33] was employed, applying a minimum average quality score threshold of 8 and a minimum read length of 360 bases across all the samples.

Taxonomic classification of the fungal reads was performed using Kraken2 2.0.8-beta [35], with the UNITE database 10.0 [36], using standard settings. The taxonomic abundance estimates generated by Kraken2 were refined using Bracken 2.6.2 [37], which employs Bayesian re-estimation to improve the estimation accuracy based on the sequence length and classification depth.

### 2.7. Data Analysis

Principal Coordinate Analysis (PCoA) was performed using Bray–Curtis dissimilarity to visualize the variation in fungal taxon composition across the six samples. PCoA plots were generated to assess the relationships and differences between the samples. Additionally, rarefaction curve analysis was conducted to assess the alpha diversity of the fungal taxa across the samples, visualizing the relationship between the sequencing depth and species richness.

### 2.8. Custom Scripts for Data Manipulation and Visualization

Custom Python scripts were developed for data manipulation and visualization, utilizing the following libraries: pandas, glob, plotly.express, plotly. graph_objects, os, re, numpy, scipy. optimize, skbio.stats.ordination, skbio.stats.distance, matplotlib, seaborn, scipy.spatial.distance, and scipy.cluster.hierarchy. The analysis utilized Bracken output files for taxonomic abundance estimation. All the bioinformatics scripts are publicly available at https://github.com/GmoreiraVet/Guitools (accessed on 12 February 2025) [38].

## 3. Results

### 3.1. Culturable Results

In total, 11 morphologically distinct fungal isolates (pure cultures) were obtained from the 123 colonies that developed from the six samples taken from the five locations. These 11 fungal isolates were classified into four genera based on their morphological characteristics. Of the 11 DNA-extracted fungi, only four were not successfully sequenced. BLASTn sequence analysis confirmed the identification of the remaining species (Table 2). All the BLASTn results had identity matches of 100.00%, except for *Aspergillus hiratsukae* (99.81%). Additionally, no colony growth was observed in the Ansião I and Moura I (1) samples.

### 3.2. Fungal Taxonomy Determination Through Metabarcoding

**Figure 2:**
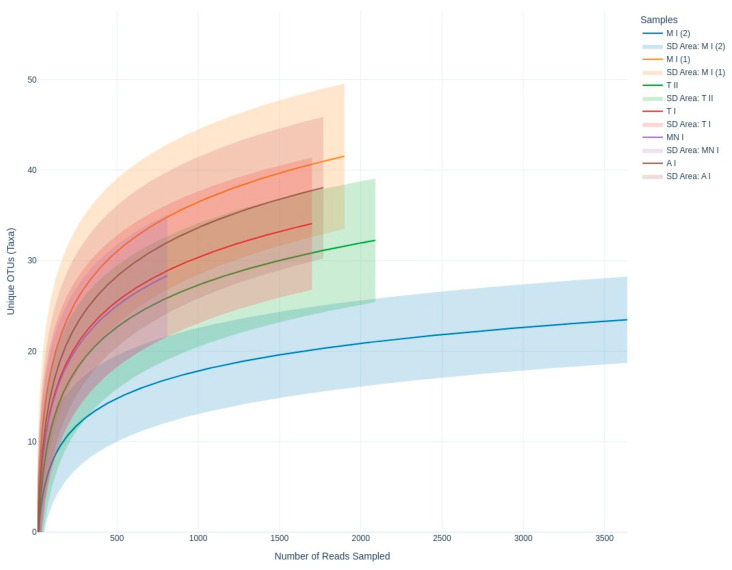
Rarefaction curves showing fungal richness as a function of sequencing depth, highlighting diversity across the sampling sites. A I: Ansião I; T I: Tomar I; T II: Tomar II; MN I: Montemor-o-Novo I; M I (1): Moura I (1); M I (2): Moura I (2).

To characterize the airborne fungal communities across the studied bat roosts, metabarcoding analysis targeting the ITS region was performed using Oxford Nanopore sequencing. This approach enabled the detection of 286 unique fungal genera across all of the sampling sites. To evaluate the diversity captured by the sequencing effort, rarefaction curves were generated for each sample (Figure 2). The curves approached an asymptote indicating that the sequencing depth was largely able to fully capture the diversity at those locations.

**Figure 3:**
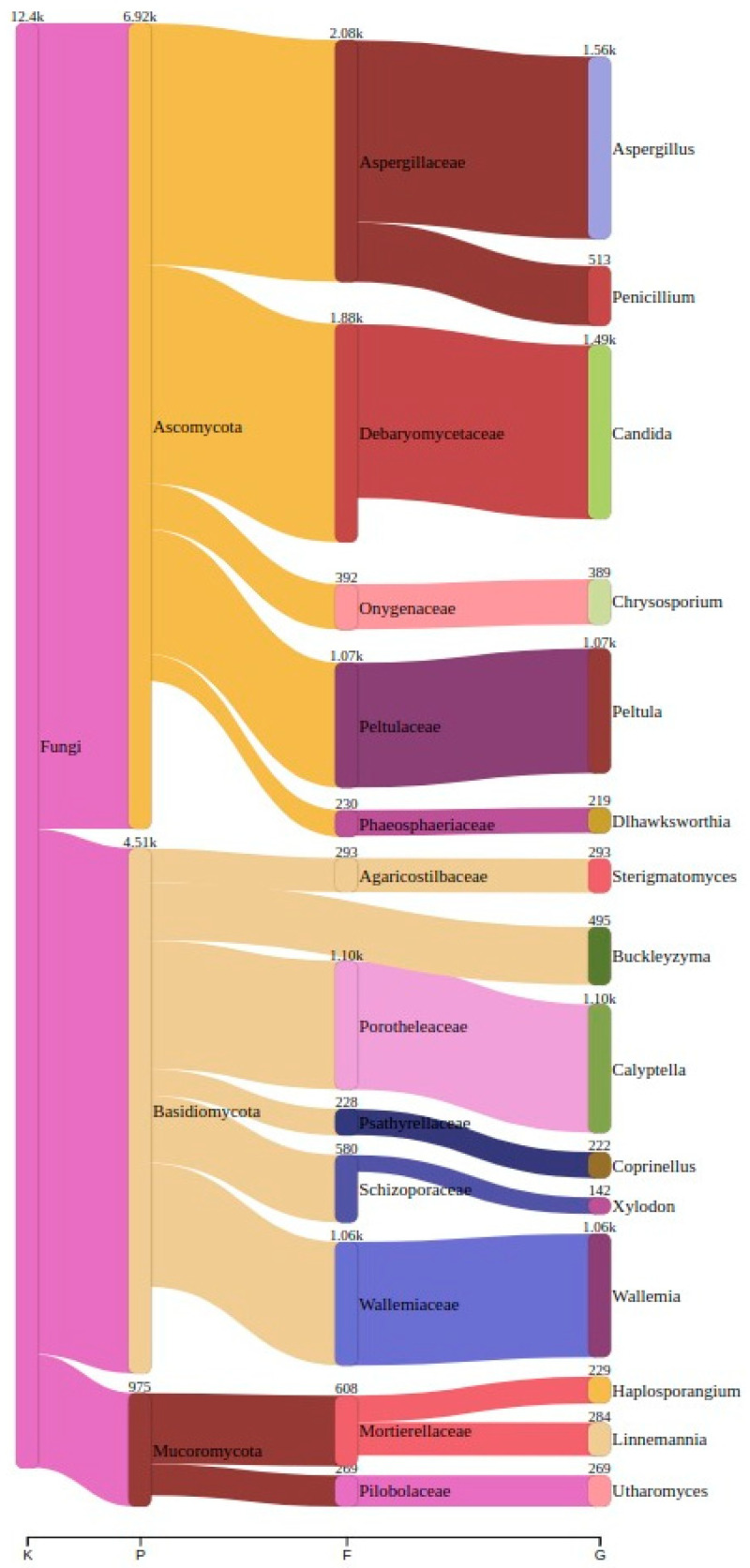
A Sankey plot showing the distribution of the top 15 genera across all the samples, illustrating the abundance of each genus among all the samples. K: kingdom; P: phylum; F: family; G: genus.

We detected a total of 286 unique genera across all the sampled sites. In the Ansião I cave, 99 genera were found, followed by 70 in Tomar I, 83 in the Tomar II cave, 119 in Moura I (1), 53 in Moura I (2), and, finally, 91 in the Montemor-o-Novo I mine. The top three genera across all the samples can be found in Table 3 and Table 4, along with their corresponding read counts, as estimated by Bracken. A comprehensive visualization of the abundance and taxonomy of the fungi across all the sampling sites is provided in Figure 3, illustrating the abundance of fungal genera across the different locations. The plot highlights the major taxonomic groups and their proportional contributions to the detected fungal community.

**Figure 4:**
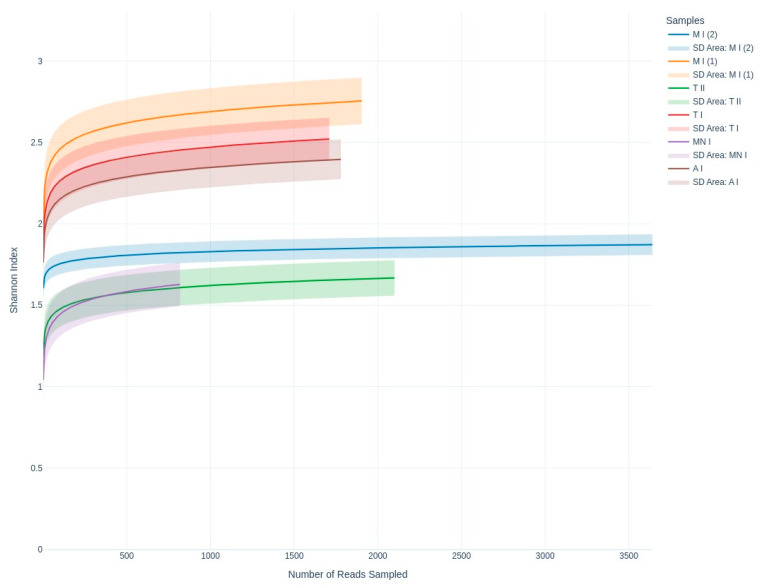
Shannon index curves illustrating fungal community diversity across the sequencing depth, emphasizing evenness and richness among the sampling sites. A I: Ansião I; T I: Tomar I; T II: Tomar II; MN I: Montemor-o-Novo I; M I (1): Moura I (1); M I (2): Moura I (2).

**Figure 5:**
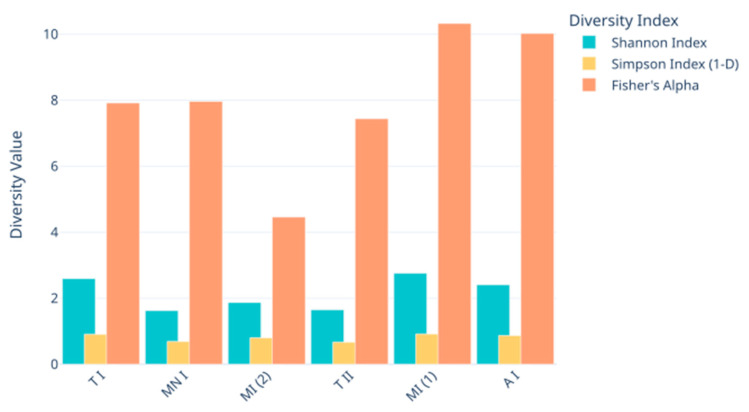
Alpha diversity metrics (Shannon Index, Simpson 1-D, and Fisher’s alpha) across the fungal metabarcoding samples. A I: Ansião I; T I: Tomar I; T II: Tomar II; MN I: Montemor-o-Novo I; M I (1): Moura I (1); M I (2): Moura I (2).

Rarefaction curve analysis was conducted to assess the alpha diversity of the fungal taxa across the samples, visualizing the relationship between the sequencing depth and species richness, utilizing the Shannon diversity index (Figure 4). Alpha diversity was further analyzed using Shannon, Simpson, and Fisher diversity indices to provide a more comprehensive assessment of the within-sample diversity (Figure 5). Samples MP1, CC, and GA exhibited higher Shannon index values, suggesting greater diversity compared to samples MP2, MN, and GN, which showed relatively lower diversity

**Figure 6:**
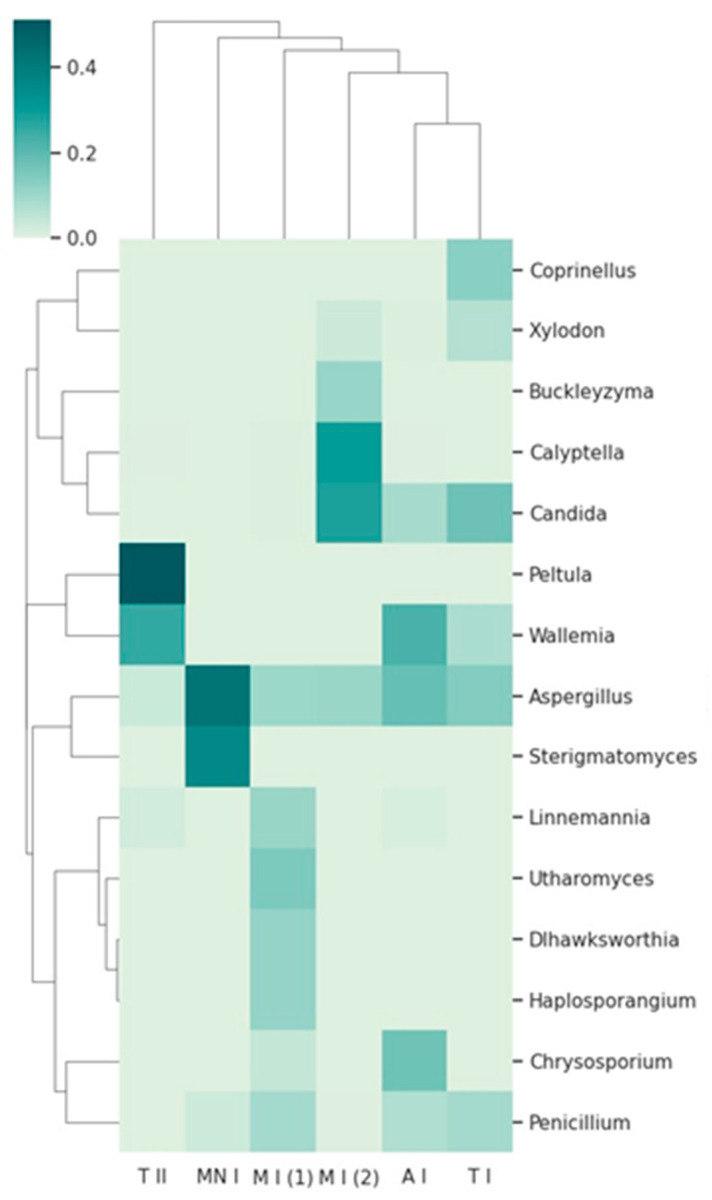
A clustergram on the taxonomic abundance at the genus level, showing the top 15 taxa across the sampling sites. The dendrograms represent hierarchical clustering of the taxa (rows) and samples (columns), based on Bray–Curtis dissimilarity. A I: Ansião I; T I: Tomar I; T II: Tomar II; MN I: Montemor-o-Novo I; M I (1): Moura I (1); M I (2): Moura I (2).

**Figure 7:**
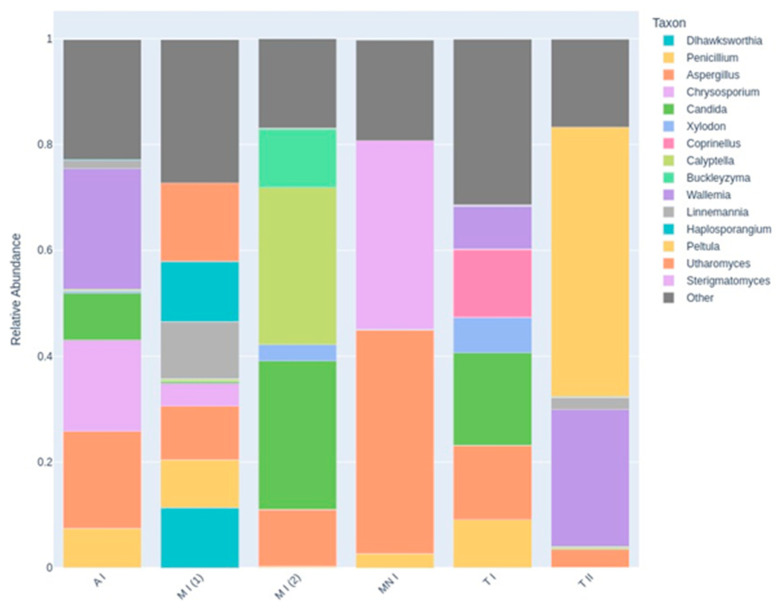
A stacked bar chart illustrating the relative abundance of the top 15 most abundant fungal genera across all the sampling sites. Each bar represents an individual air sample, labeled as follows: A I (Ansião I), T I (Tomar I), T II (Tomar II), MN I (Montemor-o-Novo I), M I (1): Moura I (1), M I (2): Moura I (2). The colored segments within each bar denote the proportional contribution of each taxon to the overall fungal community in that sample, while the “Other” category (in dark gray) aggregates all the remaining, less abundant genera that are not in the top 15.

Two visualizations were employed to illustrate the taxonomic composition across the sampling sites. The heatmap depicts the relative abundance of the top 15 taxa, with hierarchical clustering used to identify patterns of similarity among the samples (Figure 6). The stacked bar chart provides a comparative view of the relative abundance of these taxa across the sampling sites, emphasizing the distribution of dominant taxa, while incorporating an ‘Other’ category to represent less prevalent taxa (Figure 7). These visualizations offer complementary perspectives on the composition of the fungal community and its variation between the sampling sites.

To comprehensively characterize fungal diversity, we analyzed microbial communities across multiple taxonomic ranks (phyla, classes, and genera) in the studied bat roosts. At the phylum level, Ascomycota dominated all of the sites (78–92% relative abundance), followed by Basidiomycota (5–18%), consistent with oligotrophic cave environments [8,14]. Although Ascomycota and Basidiomycota were the most abundant phyla across all the samples, sequences assigned to Mucoromycota were also detected in specific locations, particularly in Moura I (2), where genera such as *Haplosporangium* were present in notable abundance. Class-level analysis revealed site-specific variations: Eurotiomycetes (e.g., Aspergillus, Penicillium) were abundant in Montemor-o-Novo I (42%) and Tomar I (38%), while Agaricomycetes (e.g., Calyptella) prevailed in Moura I (2) (51%). Notably, *Peltigerales* (lichen-forming fungi) were uniquely detected in Tomar II (51%). Genera such as *Candida* and *Wallemia* were ubiquitous, while others exhibited locality-specific patterns: *Buckleyzyma* and *Dlhawksworthia* were exclusive to Moura I (1), and *Utharomyces* was absent in Ansião I.

**Figure 8:**
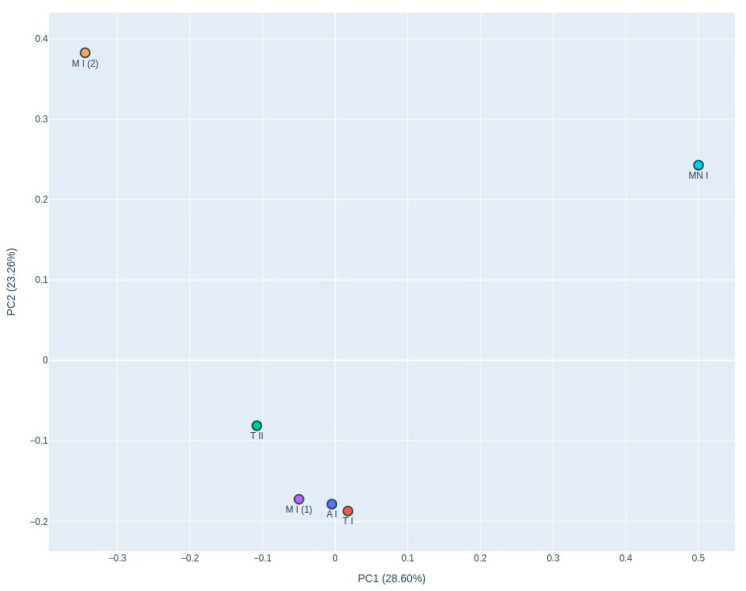
Principal Coordinate Analysis (PCoA) of the Bray–Curtis dissimilarity plot is based on beta diversity, illustrating fungal community composition across the six air samples. PC1: 28.60% variance; PC2: 23.61% variance. A I: Ansião I; T I: Tomar I; T II: Tomar II; MN I: Montemor-o-Novo I; M I (1): Moura I (1); M I (2): Moura I (2).

The PCoA graph (Figure 8), based on Bray–Curtis dissimilarity, reveals distinct clustering of the samples, with samples T I, A, M I (1), and T II grouping closely together. In contrast, M and M I (2) are positioned separately from this cluster, exhibiting divergence along the *X*-axis, although remaining relatively close along the *Y*-axis.

## 4. Discussion

Bat roosts, characterized by their extreme ecosystems, provide habitats that support the most unique fungal communities [8,10]. Despite extensive research into fungal diversity in various environments, cave air remains underexplored [11]. The present study evaluates fungal diversity in air samples from five bat roosts in six different locations, revealing a range of fungal taxa using both culture-dependent and independent methods. A total of 11 fungal taxa were isolated from culturable samples, representing genera such as *Aspergillus*, *Penicillium*, and *Chaetomium*. The fungal taxa identified using culturable methods in this study are consistent with previous reports of fungi adapted to extreme environments [39]. For instance, *Aspergillus pseudoglaucus* and *Penicillium shennongjianum* are known for their resilience and ability to thrive in nutrient-poor conditions [40,41].

The dominance of Ascomycota across all the sampling sites, followed by Basidiomycota and, in some cases, Mucoromycota, reflects the typical fungal composition of oligotrophic environments. The high presence of Eurotiomycetes (e.g., *Aspergillus*, *Penicillium*) in anthropogenic roosts, such as Montemor-o-Novo I and Tomar I, may indicate adaptation to human-impacted settings. Meanwhile, the detection of Mucoromycota in Moura I (2), particularly genera like *Haplosporangium*, suggests the presence of early-diverging lineages, possibly linked to unique microhabitat conditions in that location.

Dominant genera in the air samples included *Aspergillus*, *Candida*, and *Calyptella*, which were the most frequently detected. The PCoA revealed distinct clustering among the fungal communities in the samples. The Ansião I, Tomar I, Tomar II, and Moura I (1) samples formed a closely associated group, which indicates that these samples have similar microbial community compositions, despite their differing ecological characteristics. Ansião I and Tomar I are more open and exposed to daylight, while Tomar II and Moura I (1) are deeper and darker. This unexpected similarity suggests that factors beyond climatic conditions, such as the presence of bats, may play a key role in shaping fungal communities. In contrast, Moura I (2) and Montemor-o-Novo I exhibited unique fungal profiles, suggesting a more distinct microbial community potentially influenced by their specific environmental characteristics, including variations in nutrient availability, humidity, or visitor factors (speleologists and researchers). In addition to ecological factors, anthropogenic influences may also have contributed to shaping the fungal communities observed in certain roosts. For example, Tomar I, Moura I, and Montemor-o-Novo I are anthropogenic structures, previously subjected to human presence or modifications, and, in the case of Tomar I and Montemor-o-Novo I, both exhibited fungal profiles with dominance by opportunistic or generalist taxa, such as *Candida* and *Aspergillus*. These genera have been frequently associated with human-impacted environments and may be introduced or enriched through residual organic matter from past human use [14,18]. Moreover, anthropogenic roosts may present different microhabitats compared to natural caves, including variations in air circulation, surface material, or residual contamination.

The samples, Moura I (2) and Montemor-o-Novo I, were the furthest from the exterior, characterized by drier conditions and a high proximity to bats. This separation along PC1 and PC2, which explained 28.60% and 23.61% of the variance, respectively, reflects the influence of local environmental factors on fungal diversity. The fungal profile of Moura I (1) and Moura I (2) revealed notable differences, reflecting their distinct environmental characteristics. Although both samples were collected within the same location, they originate from different sites with contrasting conditions. Moura I (1), being more open and exposed to daylight, likely supports fungal communities adapted to fluctuating light and moisture conditions. In contrast, Moura I (2), located furthest from the exterior and characterized by drier conditions, fosters microbial communities better suited to stable conditions, which shape fungal diversity, even within broadly the same location.

In terms of the richness of the diversity across the six samples, the rarefaction curves validate the adequacy of the sequencing depth, ensuring that the observed diversity is reliable (Figure 2). Meanwhile, the alpha diversity metrics highlight diversity differences across the sampling sites, revealing the ecological or environmental factors influencing fungal communities (Figure 4 and Figure 5). While the Shannon index emphasizes both richness and evenness, Simpson’s index is more sensitive to dominant taxa, and Fisher’s alpha highlights richness alone. For instance, sample Moura I (2) displayed lower Shannon and Fisher values, indicating reduced richness and evenness. However, it presented a relatively high Simpson index, suggesting that despite a limited number of taxa, there was no strong dominance by a single genus. Conversely, samples Moura I (1) and Ansião I exhibited the highest values for Fisher’s alpha and the Shannon index, suggesting richer and more evenly distributed fungal communities, potentially reflecting more favorable or stable environmental conditions. Notably, the Simpson index remained low and relatively uniform across all the samples, reinforcing the finding that dominant taxa did not heavily skew the communities. This underlines the importance of applying a multi-index approach to comprehensively capture the complexity of the fungal community structure across varying cave environments. For example, higher diversity at Moura I (1), Tomar I, and Ansião I, all samples collected near the exterior, could reflect more favorable or variable habitats, while the lower diversity at Moura I (2), Montemor-o-Novo I, and Tomar II, collected from deeper zones, might result from environmental or resource limitations. Together, the findings emphasize the interplay between sufficient sampling and ecological factors, shaping fungal diversity in oligotrophic cave environments. As discussed earlier, although the Moura I (1) and Moura I (2) samples were both collected broadly within the same location, they were sampled from different sites with contrasting environmental conditions: Moura I (1) is more open and exposed to daylight, while Moura I (2) is located furthest from the exterior and characterized by drier conditions, highlighting the impact of microhabitat variability.

The heatmap, combined with hierarchical clustering dendrograms, for both fungal taxa and sampling sites (Figure 6), provides a comprehensive overview of the relative abundance and community structure of fungal taxa across the six sampling sites. The clustering patterns reveal distinct relationships between the sampling sites, as well as differences in taxon distribution, which may reflect underlying ecological or environmental drivers.

Some genera, such as *Buckleyzyma*, and *Calyptella*, are present only in Moura I (2). These fungi thrive in plant-rich habitats, where they can infect host plants, leading to disease development. The presence of these genera in Moura I (2) may reflect the unique surrounding environment, which includes extensive olive groves, distinguishing this location from the others, where the environmental conditions or host availability may be less conducive to their growth. *Buckleyzyma* and *Calyptella* might rely on the proximity of living plants for infection, utilizing plant tissues as nutrients and possibly contributing to plant stress or decay [42,43]. Their exclusive presence in Moura I (2) may indicate that this location supports a unique plant community or microenvironment favorable to the establishment and proliferation of these plant pathogens. These patterns point towards the existence of site-specific fungal assemblages, shaped by local ecological factors.

Additionally, the heatmap reveals a distinct dominance of *Peltula* in Tomar II, as well as the dominance of *Aspergillus* and *Sterigmatomyces* in Montemor-o-Novo I. This dominance could reflect unique ecological or environmental characteristics at Tomar II, such as the availability of specific nutrients, pH levels, or climatic conditions that favor the proliferation of certain fungi. The bat roost is located near a river in a rainy region, a setting quite distinct from Montemor-o-Novo I and Moura I, which are drier and more isolated. Such location-specific patterns underscore the potential for localized environmental factors to shape fungal community structures [44].

The presence of *Penicillium* and *Aspergillus* across most of the samples, as indicated by the heatmap, highlights their ecological adaptability and ubiquity [45,46]. These genera appear prominently in various sampling locations, suggesting that they are not limited to specific environments, but can thrive in a wide range of conditions. The heatmap shows the consistent presence of these fungi, reflecting their ability to colonize different substrates and survive in diverse ecological conditions. This widespread distribution further supports their role as generalist organisms, able to proliferate in areas with varying levels of organic matter, moisture, and temperatures [45,46]. Their adaptability and efficient spore dispersal mechanisms likely contribute to their success in such varied environments, allowing them to dominate in most of the sites surveyed.

The compositional differences between the samples are clearly illustrated in the stacked bar chart (Figure 7), which displays the relative abundance of the top 15 fungal genera across the six sampling sites. Each bar represents a distinct site, with color-coded segments indicating the proportion contributed by each genus. In Montemor-o-Novo I and Tomar II, the dominance of one or two genera is striking, as evidenced by large, nearly uniform segments occupying most of the bars. This skewed composition is indicative of low evenness and supports the lower diversity values observed in the Shannon index and PCoA analyses. In contrast, Moura I (2) presents a far more balanced community profile, with multiple genera contributing more evenly to the total composition of the community. This greater taxonomic evenness reflects higher fungal diversity, which is also consistent with its separation from MN I and T II in the PCoA plot. The high abundance of diverse genera in Moura I (2) may be ecologically linked to its location within a region known to host one of the largest bat colonies in the country, potentially enriching the local mycobiome. Additionally, the substantial size of the “Other” category in several bars (e.g., Ansião I and Tomar I) highlights a long tail of low-abundance taxa, suggesting additional, less dominant fungal diversity beyond the top genera.

The clustering observed in the heatmap and the clustering in the PCoA plot differ, which can be explained by differences in the underlying analytical methods. The heatmap focuses on a subset of relatively abundant or selected fungal taxa, highlighting differences driven by the dominant taxa. In contrast, the PCoA analysis uses the full dataset, including rarer taxa, to calculate Bray–Curtis dissimilarities, reflecting broader community-level patterns. Additionally, the PCoA captures only 52.21% of the total variance in fungal community composition, meaning that much of the variation is not represented, potentially obscuring the relationships seen in the heatmap. The heatmap allows for taxon-specific insights, which can influence site clustering. Meanwhile, PCoA condenses the data into two principal coordinates, potentially flattening subtle differences that are more evident in the heatmap’s hierarchical clustering (Figure 8).

The abundance of *Aspergillus*, *Candida*, and *Calyptella* in the cave air samples reflects the unique adaptations and ecological roles of these genera. This aligns with the adaptability of taxa such as *Aspergillus*, *Candida*, and *Calyptella* to nutrient-poor and oligotrophic environments, such as bat roosts [8,14]. *Aspergillus* species are known for their remarkable resilience, capable of surviving in nutrient-depleted environments by utilizing a wide range of organic substrates. This adaptability allows them to thrive in the oligotrophic conditions characteristic of bat roosts, wherein they contribute to nutrient cycling by decomposing organic matter brought into the ecosystem [47]. *Candida*, a genus commonly associated with opportunistic colonization, thrives in microhabitats with varying nutrient availability and moisture, which are often found in cave environments. Its versatility may also allow it to utilize specific substrates present in aerosols or biofilms within bat roosts [48]. *Calyptella*, although less commonly studied, is often associated with specialized ecological niches and may play a unique role in fungal community dynamics, potentially interacting with cave biota or contributing to biofilm formation. Together, these genera highlight the interplay of adaptability and niche specialization in shaping fungal diversity within subterranean ecosystems.

The sampling locations were all bat roosts actively frequented by bats, and previous studies have demonstrated a positive correlation between the number of bats and the concentration of airborne fungi [5]. For instance, the presence of bats significantly elevates the levels of aeromycota, as their movement, guano deposition, and shedding of skin or fur serve as nutrient sources and dispersal mechanisms for fungal spores [2,5]. These activities generate microhabitats rich in organic material, which not only support fungal growth, but also enhance spore aerosolization, significantly elevating the levels of aeromycota [5]. *Myotis myotis*, the most common bat species in Europe, has been shown to contribute to elevated fungal concentrations in underground environments, due to its large colony sizes and gregarious behavior [6]. These fungi, carried or propagated by bats, can include opportunistic pathogens that may affect both wildlife and human health. Moreover, the guano-rich environment created by bats fosters a unique microbiome, supporting the growth of fungi adapted to oligotrophic and extreme conditions typically found in bat roosts [6]. In this study, a distinct and diverse fungal community was observed particularly in Moura I (2) when compared with Moura I (1). Notably, Moura I (2) is positioned deeper within the cave system and closer to the bat aggregation site. This spatial difference may help explain the variations observed and may suggest that bat proximity and the specific microenvironment they generate, characterized by elevated humidity, guano enrichment, reduced air circulation, and limited light exposure, may be critical drivers of fungal community composition. Although no direct causal relationship can be established, these observations, together with the clustering patterns found in regard to the PCoA and diversity indices, may point to a potential ecological role of bats as contributors to microbial structuring within subterranean environments. Fungi observed in the culturable dataset were not among the most abundant in the metabarcoding analysis results, highlighting the complementary strengths of both approaches. Culture-based methods favor fast-growing or spore-forming fungi, while metabarcoding analysis captures a broader taxonomic range, including unculturable taxa [49]. The fungal diversity detected in Portuguese bat roosts underscores their ecological significance as reservoirs of unique and potentially novel species. The presence of taxa unclassifiable beyond higher taxonomic ranks points to the potential for discovering undescribed fungi in these environments. This study, the first to comprehensively assess cave air fungal diversity in Portugal, highlights the interplay between ecological and anthropogenic factors shaping these communities. It emphasizes the need for conservation measures, such as restricting access to sensitive cave areas and minimizing human impacts, to preserve native microbial diversity.

Furthermore, genera such as *Aspergillus*, *Penicillium*, *Chaetomium*, and *Candida* are commonly associated with the decomposition of organic matter, including guano, urine, fur, and skin, which are introduced into subterranean ecosystems by bats [4,14]. These mammals act as both nutrient providers and vectors, creating microenvironments that support the proliferation of opportunistic and oligotrophic-adapted fungi [5,6]. The absence of *Histoplasma capsulatum*, a well-known etiological agent of histoplasmosis, may be attributed to various factors, including the sampling strategy (air rather than guano or sediment) or limitations in the sensitivity of the detection methods [1,14]. Despite its absence, *H. capsulatum* remains of high clinical relevance in medical mycology due to its potential to cause systemic disease in humans through inhalation, especially by speleologists and researchers [26]. The identification of *Candida* spp. in the air samples may be explained by its ubiquitous nature and capacity to colonize humid environments, such as cave surfaces and biofilms. It may have been introduced by bats or speleologists and researchers [18,48]. While some *Candida* species are considered commensal, others can act as opportunistic pathogens, particularly in immunocompromised individuals. Therefore, the presence of airborne fungal genera, including potential pathogens, highlights a health risk to speleologists and reinforces the importance of implementing biosafety measures in subterranean environments [1,14].

## 5. Conclusions

This study highlights the diverse fungal communities present in Portuguese bat roosts, uncovering 286 genera through metabarcoding, alongside several culturable taxa, including *Aspergillus* and *Penicillium*. The results suggest a possible influence of environmental factors, such as nutrient availability and climatic conditions, as well as human activities, on fungal diversity and community structure. These findings underscore the ecological importance of fungi in nutrient cycling and their adaptability to oligotrophic environments. Furthermore, the identification of site-specific taxa reflects the unique ecological niches within cave ecosystems. To preserve these fragile environments and their microbial diversity, conservation strategies, such as limiting human impacts and regular monitoring, are essential to mitigate anthropogenic disturbances and ensure the sustainability of these subterranean habitats.

## Figures and Tables

**Table 1:** Bat species inhabiting each cavity studied.

Bat Species
*Myotis myotis*
*Miniopterus schreibersii*
*Rhinolophus ferrumequinum*
*Rhinolophus mehelyi*

**Table 2:** Culturable cavities fungi BLASTn (GenBank) analysis.

Fungal Taxa	Detected Sequences (Accession No.)	Bat Roosts	Query Cover (%)	Identities (%)	*E* Value	Highest Similarity Sequences (Accession No.)
*Aspergillus pseudoglaucus*	PV065740	Tomar I	100%	100.00%	0	ON645262
*Botryotrichum murorum*	PV065856	Tomar I	100%	100.00%	0	MW563925
*Aspergillus* sp. ^1^	N.A.	Tomar I	N.A.	N.A.	N.A.	N.A.
*Aspergillus pseudoglaucus*	PV069224	Tomar I	100%	100.00%	0	ON645262
*Aspergillus* sp. ^1^	N.A.	Tomar I	N.A.	N.A.	N.A.	N.A.
*Chaetomium* sp. ^1^	N.A.	Tomar II	N.A.	N.A.	N.A.	N.A.
*Chaetomium globosum*	PV066994	Moura I (2)	100%	100.00%	0	OKJ531968
*Aspergillus hiratsukae*	PV067022	Moura I (2)	100%	99.81%	0	OK94RE6FX016
*Aspergillus* sp. ^1^	N.A.	Montemor-o-Novo I	N.A.	N.A.	N.A.	N.A.
*Penicillium shennongjianum*	PV067057	Montemor-o-Novo I	100%	100.00%	0	MH862167
*Penicillium shennongjianum*	PV067130	Montemor-o-Novo I	100%	100.00%	0	MH862167

^1^ = Identified through morphological characterization only; N.A.: not applicable.

**Table 3:** Summary of unique genera and top three genera per sample.

Sample	Unique Genera	First Top Genera	Reads	Second Top Genera	Reads	Third Top Genera	Reads
Ansião I	99	*Wallemia*	405 (21.7%)	*Aspergillus*	326 (18.2%)	*Chrysosporium*	307 (16.9%)
Tomar I	70	*Candida*	299 (14.6%)	*Aspergillus*	240 (11.7%)	*Coprinellus*	220 (10.7%)
Tomar II	83	*Peltula*	1071 (50.9%)	*Wallemia*	543 (25.7%)	*Sistotrema*	91 (4.3%)
Moura I (1)	119	*Utharomyces*	281 (13.9%)	*Haplosporangium*	219 (11.3%)	*Dlhawksworthia*	217 (11.2%)
Moura I (2)	53	*Calyptella*	1079 (28.9%)	*Candida*	1024 (27.5%)	*Buckleyzyma*	405 (13.3%)
Montemor-o-Novo I	91	*Aspergillus*	346 (42.0%)	*Sterigmatomyces*	292 (35.6%)	*Filobasidium*	69 (8.4%)

**Table 4:** Top three genera across all the samples.

Top 3 Genera Across All The Samples	Reads
*Aspergillus*	1563 (12.6%)
*Candida*	1493 (12.3%)
*Calyptella*	1101 (9.1%)

## Data Availability

The data presented in this study are available on request from the corresponding author.
